# Memory and Memories: an exploratory mixed method case series study with brain injured adults and their families, using PhotoFrame Therapy and wireless digital photo frame technology

**Published:** 2012-06-15

**Authors:** Abigail Harding

**Affiliations:** Homerton Hospital Regional Neurological Rehabilitation Unit (RNRU), Homerton University Hospital NHS Foundation Trust, Homerton Row, E9 6SR, London

**Keywords:** memory, cognition, photographs, co-therapy

## Abstract

**Introduction:**

Existing research and treatment for brain injured adults uses cognitive rehabilitation to address memory deficits including compensatory and remedial techniques. Electronic devices such as the Neuropage and palm top computers are effective in compensating for memory deficits. However, these require the user to be trained and proficient in their use. This exploratory study investigates the effectiveness of pre-programmed wireless digital photo frame as a memory aid for adults with brain injury. There is evidence to suggest that people with cognitive impairment are socially isolated and digital technology has been found to provide a vehicle for social integration. A secondary consideration for the use of the digital photo frame is its potential to enhance communication and shared experiences between patient and relatives.

**Aims and objectives:**

1. To determine whether showing past photographs and prospective dates using a digital photo frame have an effect on memory function in patients with brain injury.
2. To explore the experiences of relatives in their role as co-therapists during memory sessions.

**Methods:**

Pragmatism was selected as the paradigm, as it is often associated with mixed method studies. Five participants with brain injury on a regional neurological rehabilitation unit consented. A wireless digital photo frame was set up by each bedside.

Quantitative phase:

Intervention 1: ABA’ single case experimental design. Baseline phases lasted three weeks where photo frame showed inspirational photographs, not related to the patient. Phase B the photo frame showed past photographs and prospective dates.

Intervention 2: Weekly memory sessions. Co-therapists asked patient five memory questions, relating to printed photographs using a booklet prepared for the study. One past photo, one new photo and one prospective date.

Measures used: Rivermead Behavioural Memory Test. Qualitative phase: Semi-structured interviews. Seven semi-structured interviews were conducted with the co-therapists/relatives, audio recorded and transcribed.

**Results:**

Quantitative: Descriptive statistics were used. Preliminary analysis indicates that most participants showed improvement in some of the memory sessions measures. Four of the five participants showed improvements in two or more of the memory questions presented. Areas showing most consistent improvements between subjects were working memory, autobiographical and delayed recall.

Qualitative: Thematic analysis was used.

The key themes to emerge were:

The photo frame: provided a focus for discussion between patient and relative; stimulated memories and conversation; gave the patient something to look forward to each day; improve family connections and involvement.

The memory sessions: provided a greater understanding of patient’s abilities; were challenging and stimulating for the patient.

**Conclusions:**

The use of a wireless digital photo frame used in conjunction with weekly memory sessions improves some areas of memory function in brain injured adults. Relatives experiences of being involved in rehabilitation and being able to share the experience of photographs past and present has a beneficial outcome on communication, understanding and well-being. The use of digital photo frames in memory rehabilitation warrants further research.

